# Diagnostic performance of RT-PCR-based sample pooling strategy for the detection of SARS-CoV-2

**DOI:** 10.1186/s12941-022-00501-x

**Published:** 2022-03-14

**Authors:** Miguel Hueda-Zavaleta, Cesar Copaja-Corzo, Vicente A. Benites-Zapata, Pedro Cardenas-Rueda, Jorge L. Maguiña, Alfonso J. Rodríguez-Morales

**Affiliations:** 1 Faculty of Health Sciences, Universidad Privada de Tacna, Tacna, 23003 Peru; 2 Hospital III Daniel Alcides Carrión EsSalud, Tacna, 23000 Peru; 3 Unidad de Investigación para la Generación y Síntesis de Evidencias en Salud, Universidad San Ignacio de Loyola, Lima, 15024 Peru; 4 Facultad de Ciencias de la Salud, Universidad Científica del Sur, Lima, 15024 Peru; 5 Grupo de Investigación Biomedicina, Faculty of Medicine, Fundación Universitaria Autónoma de las Américas, Belmonte, 660003 Pereira, Risaralda Colombia

**Keywords:** COVID-19, COVID-19 diagnosis, Pool testing, PCR, RT-PCR, SARS-CoV-2, Molecular diagnostic techniques, Genetic techniques, Sensitivity and specificity, Peru (MeSH)

## Abstract

**Background:**

The rapid spread of SARS-CoV-2 has created a shortage of supplies of reagents for its detection throughout the world, especially in Latin America. The pooling of samples consists of combining individual patient samples in a block and analyzing the group as a particular sample. This strategy has been shown to reduce the burden of laboratory material and logistical resources by up to 80%. Therefore, we aimed to evaluate the diagnostic performance of the pool of samples analyzed by RT-PCR to detect SARS-CoV-2.

**Methods:**

A cross-sectional study of diagnostic tests was carried out. We individually evaluated 420 samples, and 42 clusters were formed, each one with ten samples. These clusters could contain 0, 1 or 2 positive samples to simulate a positivity of 0, 10 and 20%, respectively. RT-PCR analyzed the groups for the detection of SARS-CoV-2. The area under the ROC curve (AUC), the Youden index, the global and subgroup sensitivity and specificity were calculated according to their Ct values that were classified as high (H: ≤ 25), moderate (M: 26–30) and low (L: 31–35) concentration of viral RNA.

**Results:**

From a total of 42 pools, 41 (97.6%) obtained the same result as the samples they contained (positive or negative). The AUC for pooling, Youden index, sensitivity, and specificity were 0.98 (95% CI, 0.95–1); 0.97 (95% CI, 0.90–1.03); 96.67% (95% CI; 88.58–100%) and 100% (95% CI; 95.83–100%) respectively. In the stratified analysis of the pools containing samples with Ct ≤ 25, the sensitivity was 100% (95% CI; 90–100%), while with the pools containing samples with Ct ≥ 31, the sensitivity was 80% (95% CI, 34.94–100%). Finally, a higher median was observed in the Ct of the clusters, with respect to the individual samples (p < 0.001).

**Conclusions:**

The strategy of pooling nasopharyngeal swab samples for analysis by SARS-CoV-2 RT-PCR showed high diagnostic performance.

## Background

To reduce the spread of the coronavirus disease 2019 (COVID-19) pandemic, timely diagnosis is necessary [1, 2]. The diagnosis of severe acute respiratory syndrome of coronavirus type 2 (SARS-CoV-2) is made by the real-time reverse transcription-polymerase chain reaction (RT-PCR), detection of SARS-CoV-2 antigens, or titration/detection of antibodies against it [3]; recommended among these, early diagnosis by RT-PCR [4] due to its high sensitivity and specificity, although it requires trained personnel, expensive supplies and a specialized laboratory [5].

Due to the rapid spread of the virus and the increasing demand for molecular tests to diagnose SARS-CoV-2, there has been a shortage of reagent supplies worldwide, mainly RNA extraction kits, which has caused difficulties in diagnosing suspected cases COVID-19 [6]. In this context, laboratories in different countries such as Israel, the United States, and Chile have considered grouping samples to reduce costs and speed up the diagnostic process in an environment of scarce resources [7–10].

Clustering combines individual patient samples (e.g., ten) into one block and analyses the group as a single sample. If the cluster evaluation is negative, all individual samples are negative; Likewise, if it presents a positive result, at least one of the individual samples is positive, so they must be individually analyzed to determine which samples are positive [11, 12].

Due to the precarious health system that various Latin American countries have, efficient strategies need to be implemented for the early diagnosis of COVID-19. The pooling of RT-PCR could meet this goal. Therefore, the objective of this study was to evaluate the sensitivity and specificity of the strategy of pooling nasopharyngeal swab (NS) samples for the detection of SARS-CoV-2 by RT-PCR.

## Methods

### Study design and population

We carried out a cross-sectional study of diagnostic tests to evaluate the diagnostic performance of the strategy of grouping NS samples to detect SARS-CoV-2 employing RT-PCR, compared with the individual RT-PCR of samples of NS. NS were collected during the period from september to december 2020.

**Fig. 1:**
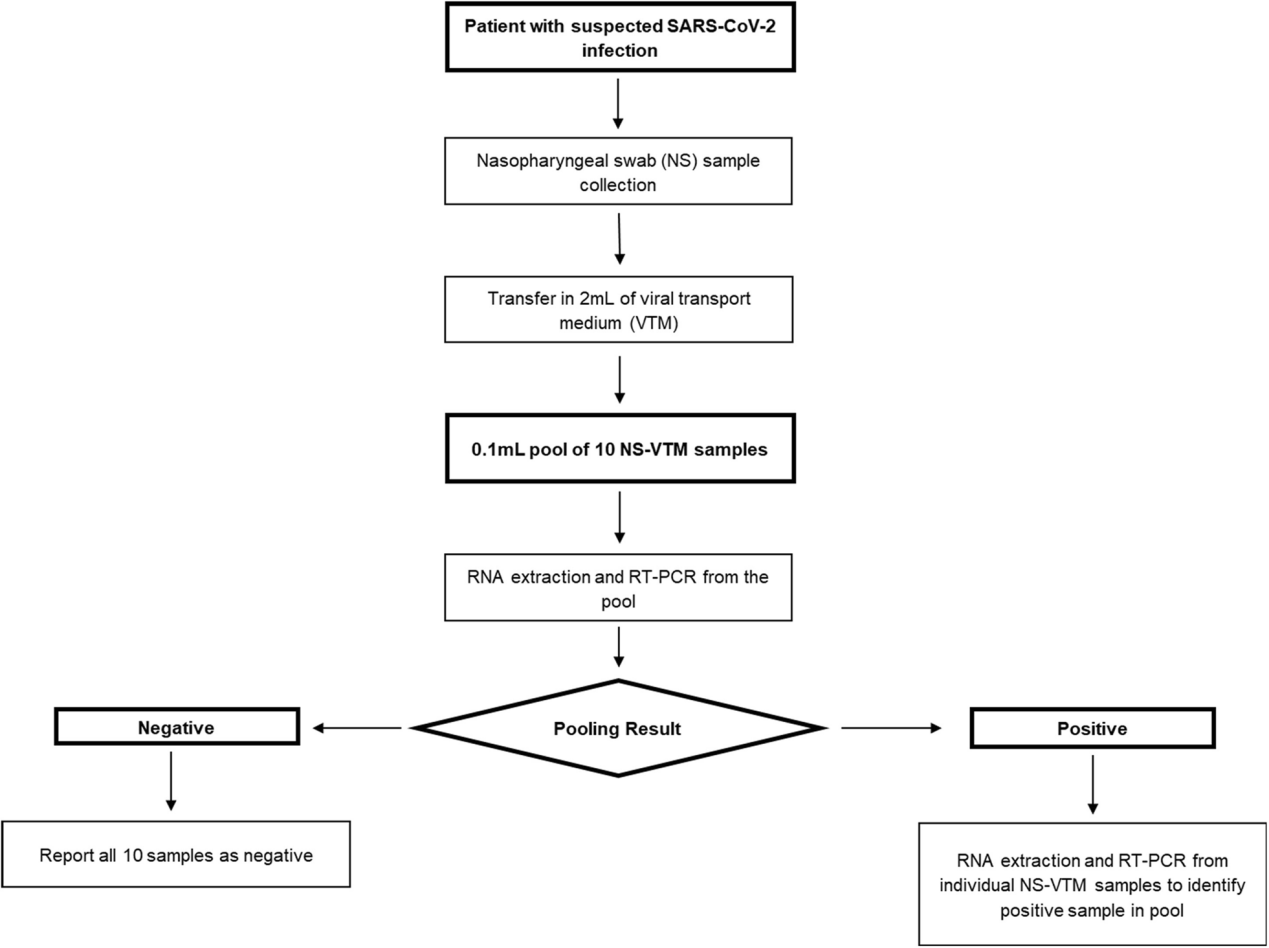
Distribution of nasopharyngeal swab samples for subsequent analysis

People diagnosed at Hospital III Daniel Alcides Carrión and Hospital Base Hipolito Unanue, located in the province and region of Tacna in Peru, were evaluated. Participants attended to rule out SARS-CoV-2 infection by taking an NS sample that RT-PCR then analyzed; patients with symptoms of up to 7 days of evolution and who also signed the informed consent were included in the study (Fig. 1).

### Transportation, handling, and pooling of samples

**Fig. 2:**
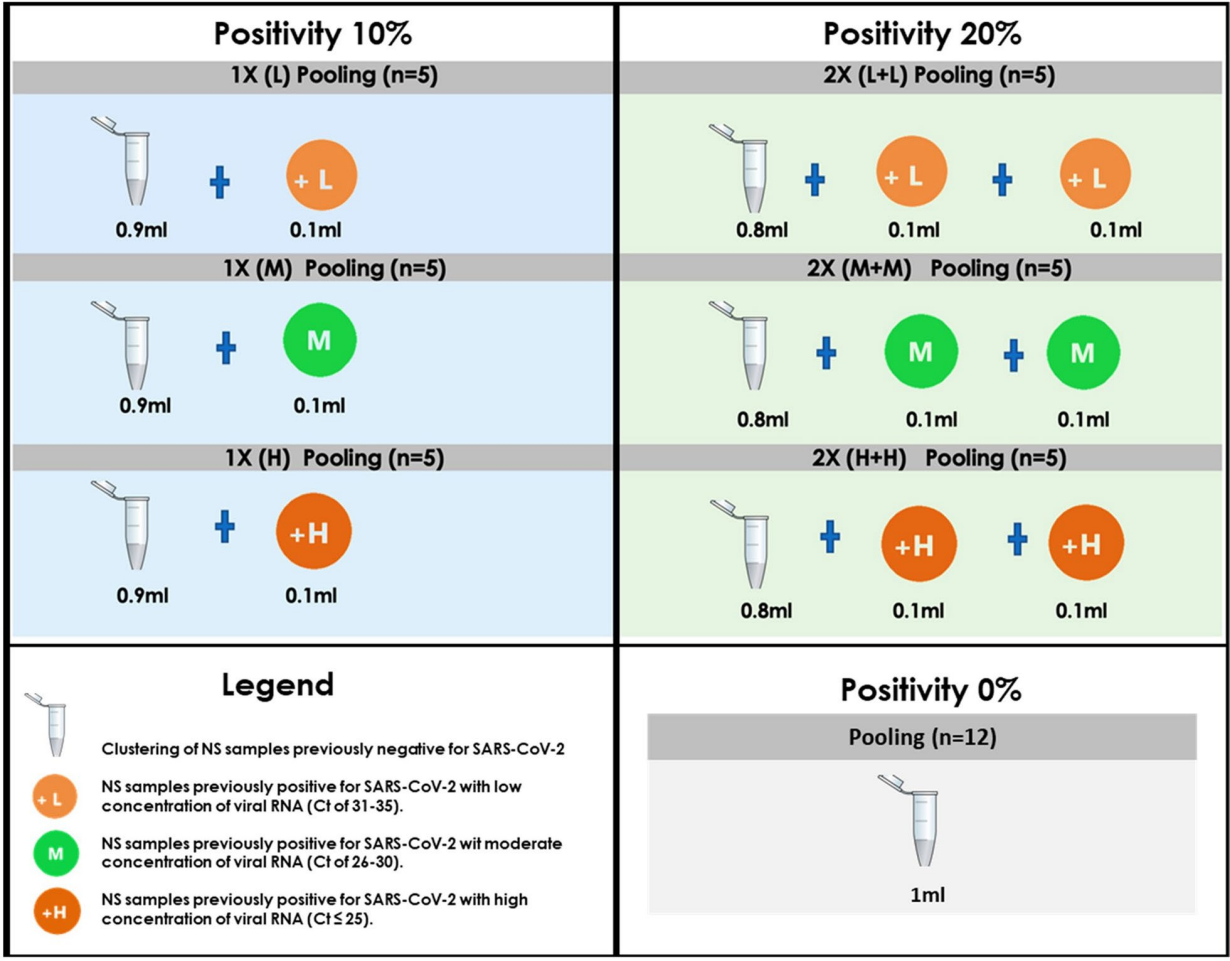
Pooling of nasopharyngeal swab samples according to positivity and concentration of SARS-CoV-2 viral RNA

After taking the individual NS samples, they were transferred in 2 mL of the viral transport medium (VTM). Later, they were analyzed by RT-PCR of the SARS-CoV-2 virus following the manufacturer's instructions [13]. Forty-five of these samples were positive and were classified according to their Ct values as high (H: ≤ 25), moderate (M: 26–30), and low (L: 31–35) concentration of viral RNA [14]; the rest (375) had a negative result. From these NS samples (positive and negative), 42 clusters were formed, each containing ten samples. These pools contained a total of 1.0 mL of NS-VTM (0.1 mL for each sample) and were categorized into three groups. Fifteen clusters were formed from the combination of 0.1 mL of NS-VTM from a positive sample for SARS-CoV-2 with nine negative samples, each with 0.1 mL of NS-VTM (total, 0.9 mL), simulating a positivity of 10%. These first 15 clusters were subdivided into 5 clusters with a high, moderate, and low concentration of viral RNA, respectively. The second block of 15 clusters was formed considering two positive samples for SARS-CoV-2, each with 0.1 mL of NS-VTM (total, 0.2 mL), with eight negative samples, each with 0.1 mL of NS-VTM (total, 0.8 mL), simulating a positivity of 20%. This second set of 15 clusters was subdivided similarly to the first (high, moderate, and low). Finally, the third group evaluated 12 clusters formed with ten negative samples for SARS-CoV-2, each with 0.1 mL of NS-VMT (total, 1.0 mL). These distributions were made using the study by Wacharapluesadee et al. [14] (Fig. 2).

### SARS-CoV-2 detection

RNA extractions were performed from 100 μL of VTM pooled using the RADI COVID-19 Detection Kit from KH medical Co. For the RT-PCR, the SD Biosensor brand Standard M nCoV real-time PCR kit was used, which detects the RdRp and E genes of the virus, following the procedures specified by the manufacturer [13]. The reaction was run in the QIAGEN Model RotorGene™ brand real-time Thermal Cycler. The individual and group samples were considered positive when the SARS-CoV-2 target (RdRp gene and E gene) had a Ct < 36; otherwise (Ct ≥ 36), they were deemed to be negative, as indicated by the instructions for use [13].

### Statistic analysis

All data were analyzed using the Stata version 16 statistical program (StataCorp., Texas, USA). The individual and grouping Ct values were presented in their median and interquartile range (IQR) due to their non-symmetric distribution. The bivariate analysis was performed using the Wilcoxon statistical test of signs and ranges to compare the median of the Ct values of the samples before being grouped with the Ct values after the grouping.

We evaluate the NS sample grouping strategy using the RT-PCR test results of individual samples were taken as a reference. First, we calculated the area under the ROC curve (Receiver Operating Characteristic) the Youden index, as well as sensitivity, specificity, likelihood ratio (LR), positive and negative predictive value with their respective global 95% CI and by subgroups according to their Ct values that were classified as high (H: ≤ 25), moderate (M: 26–30) and low (L: 31–35) concentration of viral RNA [14]. However, the positive and negative LR could not be calculated in all the subgroups since the sensitivity, and specificity parameters in some of these reached 100%, generating a calculation of LR that was not estimable.

### Ethical aspects

This research adheres to the Helsinki standards for research on human subjects. The protocol was approved by the institutional research ethics committee of the Private University of Tacna (Registration Code: 045-FACSA-UI) and by the research committees of the Hospitals: Daniel Alcides Carrión EsSalud-Tacna and Hipolito Unanue-Tacna. Likewise, it was entered with code EI00000001461 to the national registry of health research projects (PRISA) developed by the National Institute of Health of Peru (INS-Peru). Informed consent was requested from all patients who participated in the research, providing their nasopharyngeal swab samples for RT-PCR analysis.

The Universidad Privada de Tacna funded the study. Support was received from the Regional Directorate of Health of Tacna (DIRESA-Tacna) for molecular analysis through its molecular biology laboratory.

## Results

All pools that contained positive individual samples managed to amplify the RdRp gene marker, even those pools that contained a single positive sample with Ct between 30 and 35. In 41 of 42 clusters (97.6%), similar results were observed in the individual samples analyzed (29 clusters that included at least one positive sample and 12 negative clusters). Only one of 42 clusters presented a Ct ≥ 36 (Ct: 36.86), this being considered negative and discordant with the individual positive sample (Ct: 32.28).

The Youden index had a value of 0.97 (95% CI, 0.90–1.03). The sensitivity and specificity of the clusters was 96.67% (95% CI, 88.58–100%) and 100% (95% CI, 95.83–100%) respectively. Likewise, the value of the area under the ROC curve was 0.98 (95% CI 0.95–1.0) (Table 1). In the stratified analysis of the groups containing positive samples with Ct ≤ 25, the sensitivity and specificity found was 100% (95% CI; 90–100%) and 100% (95% CI; 95.83–100%), respectively. While with the groupings containing positive samples with Ct ≥ 31, the sensitivity and specificity found was 80% (95% CI; 34.94–100%) and 100% (95% CI; 95.83–100%), respectively.

**Table 1:** Evaluation of the strategy for pooling NS samples for analysis by RT-PCR

Analysis	Total pooling		Pooling Ct ≤ 25		Pooling Ct: 26–30		Pooling Ct: 31–35	
	Value	95% CI	Value	95% CI	Value	95% CI	Value	95% CI
AUC-ROC	0.98	0.95–1.00	1.00	100–100	1.00	100–100	0.90	0.70–1.00
Sensitivity (%)	96.67	88.58–100	100	90–100	100	90–100	80	34.94–100
Specificity (%)	100	95.83–100	100	95.83–100	100	95.83–100	100	95.83–100
Predictive value + (%)	100	98.20–100	100	90–100	100	90–100	100	87.50–100
Predictive value – (%)	92.31	73.98–100	100	95.83–100	100	95.83–100	92.31	73.98–100
Likelihood ratio +	NE	NE	NE	NE	NE	NE	NE	NE
Likelihood ratio −	0.03	0.00–0.23	NE	NE	NE	NE	0.20	0.03–1.15
Youden index	0.97	0.90–1.03	1.00	100–100	1.00	100–100	0.80	0.45–1.15

*AUC* Area down the curve, *ROC* receptor operating characteristic, *NE* not estimable, *NS* nasopharyngeal swab, *RT-PCR* reverse transcriptase-polymerase chain reaction. The likelihood ratio could not be estimated in all groups and subgroups since the sensitivity and specificity had values of 100%

In the clusters, a higher median was observed in the Ct of the RdRp gene for the Ct of the individual samples; this difference was statistically significant (p < 0.001) (Table 2).

**Table 2:** Difference in cycle threshold values of individual and grouped samples according to the scenario of 10% and 20% positivity for SARS-CoV-2

Variable	Individual samples	Pooled samples	P value
Ct Total (n = 45)*	28.47 (20.76–31.36)	29.88 (23.77–33.74)	< 0.001^a^
Pooling 1/10 (10%)			
Ct: ≤ 25 (n = 5)*	18.46 (17.86–18.82)	20.22 (20.02–22.26)	0.043^a^
Ct: 26–30 (n = 5)*	29.37 (29.22–29.66)	32.87 (31.75–33.93)	0.042^a^
Ct: 31–35 (n = 5)*	32.15 (31.94–32.28)	34.67 (33.67–34.81)	0.043^a^
Pooling 2/10 (20%)			
Ct: ≤ 25 (n = 10)*	20.58 (17.31–21.14)	22.68 (21.20–23.77)	0.041^a^
Ct: 26–30 (n = 10)*	28.21 (27.56–28.44)	28.60 (28.56–28.61)	0.138^a^
Ct: 31–35 (n = 10)*	31.40 (31.19–32.36)	33.74 (33.20–34.85)	0.125^a^

*Median and interquartile range, ^a^Wilcoxon statistical test of signs and ranges. *Ct* cycle threshold

## Discussion

In this study, the strategy of grouping NS samples for analysis by RT-PCR showed high sensitivity and specificity after being compared with the reference test (RT-PCR of individual samples). Nevertheless, there was a decrease in sensitivity by dilution in pools containing a single positive sample with low SARS-CoV-2 viral load (Ct ≥ 31). These data suggest that NS samples, especially from patients in the first days of illness (higher viral load) [15], could be pooled and analyzed for SARS-CoV-2 virus by RT-PCR with detection levels similar to those obtained by processing each sample individually.

A vital consideration in the strategy of grouping NS samples is the possibility of false-negative results, this due to the dilution that occurs when grouping the samples, mainly in those with low viral load (Ct ≥ 31) [16], as reported in this study, in which one of the clusters had a discordant (negative) result with the positive sample is included. This finding is consistent with other research, which reports that, when pooling 36 to 50 samples, the proportion of false negatives rises considerably [17, 18]. Due to this, it is proposed that the most appropriate use of grouping would be using 5 to 10 samples per group since the sensitivity would be greater than 90% [14, 19].

Gremmels et al. [20] evaluated the Panbio™ COVID-19 Rapid Antigen Test (Abbott) compared to RT-qPCR in symptomatic patients in a medium and high endemicity scenario of sensitivity between 72.6 to 81% was observed and a specificity of 100%. However, when the Ct was ≥ 32, the sensitivity decreased dramatically from 0 to 21%, representing a high rate of false negatives that could have a detrimental impact on the transmission of SARS-CoV-2 in the community. Furthermore, in our study, the sensitivity of the clusters that contained at least one positive sample with high Ct showed a possible better performance for the diagnosis of SARS-CoV-2, which also decreased.

In terms of efficiency, grouping samples can reduce the burden of laboratory material and logistics resources by up to 80% [14, 16]. More important, however, is the potential to massively increase the number of individuals tested using the same number of reagents (test kits). This aspect is a critical advantage given the shortage of test kits, especially in low- and middle-income countries [16]. However, if the stakeholders want to implement this strategy, they should have some considerations. First of all, this strategy would be helpful in populations where the positivity rate is low (less than 10%) [14], such as for routine monitoring of workgroups (health personnel, military units, and factory workers), where diagnosing even one positive person requires quarantine of the entire group to prevent further spread in the community [19]. On the other hand, in populations where the positivity rate is high, it may not be adequate since it would give more positive groups that will affect the response time and require a more significant amount of tests, consuming more resources [12]. Another point to consider is the time of illness of the patients, which is probably more helpful in patients in the first days of illness since a higher viral load could be found [15].

This study has certain limitations. The main one was the limited number of clusters due to the limited budget available, which could generate imprecision in the reported results and, in turn, did not allow more clusters with high Ct, where the increase in Ct could affect the sensitivity of the proof. Second, PCR quantification of the SARS-CoV-2 viral load was not performed in the individual samples or clusters, which would have helped accurately measure the variation in a viral load. On the other hand, the bias attributed to the disease stage in which the study participants were found must be taken into account. The disease stage could have affected the viral load and, therefore, the detection of SARS-CoV-2. More studies are needed with a more significant number of clusters analyzed, especially in specific scenarios, where the cost–benefit impact of this strategy can be evaluated.

## Conclusions

In conclusion, this study showed that the strategy of pooling up to 10 NS samples for SARS-CoV-2 RT-PCR analysis has a high diagnostic yield, but it must be taken into account that in patients with low viral load (as in later stages of the disease) pooling of samples can greatly decrease in sensitivity. This finding is an alternative to the existing ones for diagnosing SARS-CoV-2 in low to the very low prevalence of the disease. Furthermore, it could reduce laboratory resources and, in turn, increase the detection of patients with COVID-19.

## Data Availability

Upon request.
